# hsa_circ_0129047 Upregulates LYVE1 to Inhibit Hepatocellular Carcinoma Progression by Sponging miR-492

**DOI:** 10.1155/2023/6978234

**Published:** 2023-09-30

**Authors:** Zhenzhen Feng, Jiyuan Wu

**Affiliations:** Department of Infectious Diseases, Affiliated Puren Hospital of Wuhan University of Science and Technology, Wuhan 430080, Hubei, China

## Abstract

Compelling evidence indicates the regulatory role of circular RNAs in cancers, including hepatocellular carcinoma (HCC). Our study aimed to elucidate the regulatory function of circ_0129047 in HCC progression. A reverse transcription-quantitative polymeric chain reaction was conducted to detect the expression of circ_0129047, lymphatic vessel endothelial hyaluronan receptor-1 (LYVE1), and miR-492 in HCC tissues and cells. The characteristics of circ_0129047 were determined by evaluating the nuclear and cytoplasmic fractions and by RNase R digestion assays. The cell counting kit-8 assay, scratch wound, and transwell invasion assays were used to examine the effects of circ_0129047 overexpression, miR-492 mimic, and LYVE1 overexpression on the proliferation, migration, and invasion abilities of HCC cells in vitro. A mouse xenograft model was also established. The relationship between miR-492 and circ_0129047 or LYVE1 was clarified using luciferase reporter and Argonaute-2 RNA immunoprecipitation assays. We found that circ_0129047 and LYVE1 were poorly expressed in HCC tissues and cells, whereas miR-492 was upregulated. Overexpression of circ_0129047 inhibits HCC cell proliferation, migration, and invasion and delays in vivo tumor growth. Furthermore, circ_0129047 sponged miR-492, and 3′UTR LYVE1 was a direct target of miR-492. Additionally, LYVE1 overexpression reduced the oncogenic activity of the miR-492 mimic, whereas the miR-492 mimic abolished the antimigratory, antiproliferative, and anti-invasive effects of circ_0129047 overexpression in HCC cells. These data suggest that circ_0129047 exerts a tumor-suppressive role in HCC by sponging miR-492 away from LYVE1 and that the circ_0129047/miR-492/LYVE1 axis may be a promising target for HCC treatment.

## 1. Introduction

Following lung and colorectal cancers, liver cancer is listed as the third leading cause of global cancer-associated fatalities [1]. In 2021, liver cancer was responsible for 830,180 deaths [1]. Despite the reduction in the incidence of liver cancer, it is still the sixth most prevalent malignancy, accounting for 906,000 newly diagnosed patients in 2020 [2]. Hepatocellular carcinoma (HCC), which accounts for between 75% and 85% of liver cancer cases, is the most common subtype of liver cancer [3]. Epidemiological investigation has shown that HCC is more common in patients suffering from hepatitis B virus and hepatitis C virus infections [4]. Thus, understanding the molecular basis of HCC may aid in its treatment.

Circular RNAs (circRNAs) are endogenous noncoding RNAs that are characteristic covalent closed-loop structures formed by joining the 3′ and 5′ ends [5]. Their special structures confer structural stability compared to those of their linear counterparts. Although they do not encode proteins, circRNAs affect gene expression through multiple mechanisms [5]. Cytoplasmic circRNAs are characterized by competing endogenous RNA (ceRNA) activity, wherein they sponge microRNAs (miRNAs). Thus, circRNAs shape protein production through complementary base-pairing recognition by miRNAs [6]. Currently, many cytoplasmic circRNAs are known to have a role in HCC progression [7]. For instance, the ceRNA activity of circRNA-SORE against miR-103a-2-5p and miR-660-3p hyperactivates the oncogenic Wnt/-catenin pathway, leading to sorafenib resistance in HCC [8]. Another circRNA, cSMARCA5, has been shown to inhibit the growth and migration of HCC and is a promising therapeutic target in HCC [9]. CircMET is an oncogenic circRNA in HCC that enhances the immunosuppressive tumor microenvironment through the miR-30-5p/snail/DPP4 axis [10]. Although ceRNA activity of circ_0129047 has been demonstrated in lung adenocarcinoma [11], its role in HCC remains unclear.

Similar to circRNAs, miRNAs are a group of noncoding transcripts. Despite being only approximately 20 nucleotides long, miRNAs can crosstalk with circRNAs, actively fine-tune the expression of their target genes, and influence cellular behavior in a step-wise manner [12]. Owing to their high stability, miRNAs may be attractive biomarkers for clinical outcomes in evaluating cancer progression [13]. For example, the high expression of miR-3682-3p is upregulated in HCC and linked to poor prognosis in HCC patients [14]. MiR-200b-3p negatively regulated angiogenesis and attenuated cancer metastasis in HCC [15]. Similarly, the abnormal expression of miR-492 has been detected in hepatic cancer and contributes to HCC pathology [16]; however, its detailed molecular mechanism remains unclear.

The lymphatic vessel endothelial hyaluronan receptor-1 (LYVE1) gene is located on chromosome 11p15.4 and comprises six exons. As a type I integral membrane glycoprotein, it is a widely used signature of lymphatic endothelial cells, which are critical for tumor metastasis [17]. Increasing evidence has demonstrated that high LYVE1 expression is associated with unfavorable clinical outcomes [17]. Pharmacological inhibition of LYVE1 suppresses the vasculature content and tumor angiogenesis [18]. However, the downregulation of LYVE1 was observed in HCC and was associated with favorable clinical outcomes [19]. However, the regulatory mechanisms in HCC have not yet been elucidated.

In this study, we constructed a ceRNA network comprising circ_0129047, miR-492, and LYVE1. Therefore, we hypothesized that circ_0129047 participates in the progression of HCC. Using gain-of-function assays, we elucidated the role of circ_0129047 in vitro and in vivo. Further mechanistic investigations were conducted to validate the ceRNA activity of circ_0129047. Our findings provide a theoretical basis for the potential transferability of circRNAs to clinical practice.

## 2. Methods

### 2.1. Clinical Specimen

We obtained 32 matched fresh primary HCC tissues along with corresponding tumor-free tissues from the patients who underwent surgical resection at Wuhan University of Science and Technology's Affiliated Puren Hospital. The Affiliated Puren Hospital of Wuhan University of Science & Technology's Ethics Committee provided consent to our request. The enrolled patients had not received any treatment before surgery. The pathology of the samples was confirmed by pathologists after surgery. Samples were stored at −80°C until further use.

### 2.2. Cell Culture

A human liver cell line (THLE2) and two HCC cell lines (Hep 3B and SNU-182) were purchased from the American Type Culture Collection (ATCC), Manassas, VA, USA. The third HCC cell line, Huh7, was purchased from ProCell (China). THLE2 cells were cultured in Bronchial Epithelial Cell Growth Medium (BEGM; LONZA, USA), Huh7 cells in Dulbecco's Modified Eagle Medium (DMEM; ProCell, China), Hep 3B cells in Eagle's Minimum Essential Medium (MEM, ProCell), and SNU-182 cells in Roswell Park Memorial Institute (RPMI)-1640 medium (ProCell). All media were supplemented with 10% fetal bovine serum (FBS; Procell) and 1% penicillin/streptomycin (ProCell). Additionally, all cell lines were grown at 37°C with 5% CO_2_.

### 2.3. Cell Transfection and Infection

pcDNA-Circ_0129047 (OE-circ), pcDNA-LYVE1-overexpressing vectors (OE-LYVE1), miR-492 mimics, and their matched negative controls (NCs) were purchased from GenePharma (China). Upon 80% confluence, Huh7 and Hep 3B cells were transfected with the aforementioned products using Lipofectamine 2000 (ThermoFisher, USA). Gene expression was quantified by reverse transcription-quantitative polymeric chain reaction (RT-qPCR) at 48 hr posttransfection.

Circ_0129047-overexpressing lentivirus (OE-circ) and empty lentivirus (OE-NC) particles were purchased from GenePharma. The lentiviral particles (multiplicity of infection = 0.5) were infected with Huh7 and Hep 3B cells for 48 hr. The infection efficiency was validated using RT-qPCR.

### 2.4. RT-qPCR

A total RNA isolation kit (TIANGEN, China) was used for RNA extraction from tissues and cells. Using the Quantitect Reverse Transcription Kit (Qiagen, China), 2 *µ*g of total RNA was subjected to reverse transcription to cDNA. Subsequent amplification and quantification of cDNA were conducted using Power Green qPCR Mix (Takara Bio, China). Target gene fold changes were calculated using the 2^−*ΔΔ*Ct^ technique and normalized to the glyceraldehyde-3-phosphate dehydrogenase (GAPDH) or U6 control. Primers used are listed in Table 1.

### 2.5. Separation of Nuclear and Cytoplasmic Fractions and RNAse Treatment

Nuclear and cytoplasmic fractions of Huh7 and Hep 3B cells were prepared using a Nuclear/Cytosol Fractionation Kit (Phygene, China) according to the manufacturer's instructions. RT-qPCR was conducted to determine the circ_0129047 expression in the nucleus and cytoplasm.

For the RNase treatment assay, RNAs isolated from Huh7 and Hep 3B cells were treated with RNase A (Novo Biotechnology Co., Ltd., China). After 1 hr, RT-qPCR was conducted to determine the circ_0129047 expression. Linear gene circ_0129047 (LIFR) was used as an NC.

### 2.6. Western Blot

Radioimmunoprecipitation assay lysis buffer (Beyotime, China) was used to extract the total protein from Huh7 and Hep 3B cells, followed by protein quantification using a bicinchoninic acid protein quantitative kit (Beyotime, China). Subsequently, the 20 *µ*g protein samples were separated by 12% sodium dodecyl–sulfate-polyacrylamide gel electrophoresis and then transferred onto polyvinylidene difluoride membranes. After blocking with 5% nonfat milk at room temperature for 1 hr, the membranes were incubated with the primary antibodies, including anti-LYVE1 (Cat#: CSB-PA897574ESR1HU, 1 : 1000, CUSABIO, China) and anti-GAPDH (Cat#: CSB-PA00025A0Rb, 1 : 1000, CUSABIO) overnight at 4°C. The next day, the membranes were incubated with the horseradish peroxidase-conjugated antirabbit secondary antibody (Cat#: CSB-PA00120E1Rb, 1 : 5000, CUSABIO) and were visualized using BeyoECL Plus (Beyotime) at 37°C, and the protein bands were photographed.

### 2.7. Cell Counting Kit-8 (CCK8) Assays

CCK8 (Beyotime) was used to measure cellular proliferation (Beyotime). Huh7 and Hep 3B cells (3,000 cells per well) that were 80% confluent were seeded in 96-well plates and cultured for 0, 24, 48, or 72 hr. This was followed by an incubation with 10 *µ*L of CCK8 reagent for 2 hr. After 2 hr, the plates were read with a microplate reader (Sigma, USA) at 450 nm.

### 2.8. Scratch Migration Assay

Huh7 and Hep 3B cells were seeded into 6-well plates. When cells were grown to 80%–90% confluence, a wound scratch field was made in each well using a sterile 200 *μ*L pipette tip. The scratched cells were cleaned with phosphate-buffered saline. Cells were imaged at 0 and 24 hr of culturing to assess wound closure. The migration rate of the cells was calculated as follows: migration rate (%) = (wound width at 0 hr – wound width at 24 hr)/wound width at 0 hr × 100.

### 2.9. Transwell Invasion Assay

To assess the cell invasion, we prepared the inserts with coated matrigel. Subsequently, Huh7 and Hep 3B cells (5 × 10^4^) suspended in a 200 *µ*L medium without FBS were added to the interior of the inserts, and the inserts were placed on a 24-well companion plate. To each well of the plate, we added 600 *µ*L of medium containing 10% FBS. After 24 hr, the inserts were fixed with 500 *μ*L of 4% paraformaldehyde for 15 min and subjected to staining with 500 *μ*L of crystal-violet solution for another 15 min. After scrubbing the non-invading cells with cotton-tipped swabs, the remaining cells were counted and imaged under a microscope.

### 2.10. Xenograft Assay

Nude mice that were 6 weeks old were purchased from Wuhan University's Experimental Animal Center (China). The Affiliated Puren Hospital of Wuhan University of Science and Technology's Institution Animal Ethics Committee approved the use of animals in this study. The mice were kept under specific pathogen-free conditions, receiving a 12-hr light/12-hr dark cycle. After 1 week, Hep 3B cells transfected with the circ_0129047-overexpressing lentiviral plasmid or control lentivirus were subcutaneously administered to mice. Vernier calipers were used to measure the size of the tumors every week. Mice were euthanized by CO_2_ inhalation and cervical dislocation 5 weeks later. The tumors were weighed after dissection.

### 2.11. Luciferase Reporter Assay

All the luciferase reporter vectors were constructed by GenePharma (Shanghai, China). In particular, the pGL3 luciferase vectors were inserted into the wild-type (WT) putative sequence of circ_0129047 or LYVE1 3′UTR and their corresponding mutant (MUT) sequence, and the recombinant vectors were named circ_0129047-WT, circ_0129047-MUT, LYVE1 3'UTR WT, and LYVE1 3′UTR MUT. The resulting vectors were cointroduced into Hep 3B and Huh7 cells with the miR-492 mimic or mimic NC. After 48 hr, luciferase activity was measured using the Dual-Lumi Luciferase Reporter Gene Assay Kit (Beyotime, China) and normalized to the Renilla luciferase activity.

### 2.12. RNA Immunoprecipitation

Hep 3B and Huh cells were lysed and sonicated. The prepared cell lysates were incubated with the beads preattached with anti-AGO2 or anti-IgG at 4°C overnight. The immunoprecipitated complex was isolated and subjected to RNA isolation using the Total RNA Isolation Kit (TIANGEN, China). The expression levels of circ_0129047 and miR-492 were quantified using RT-qPCR.

### 2.13. Statistical Analysis

Analyses were conducted using the GraphPad Prism software (GraphPad, USA). Student's *t*-test or one-way analysis of variance with Tukey's post hoc test was used to analyze the data from two and multiple groups, respectively. Pearson's correlation analysis was conducted to determine the correlation between miR-492 and circ_0129047 or LYVE1 expression. Statistical significance was defined as *P* < 0.05.

## 3. Results

### 3.1. Circ_0129047 Expression Decreased during HCC Progression

**Figure 1:**
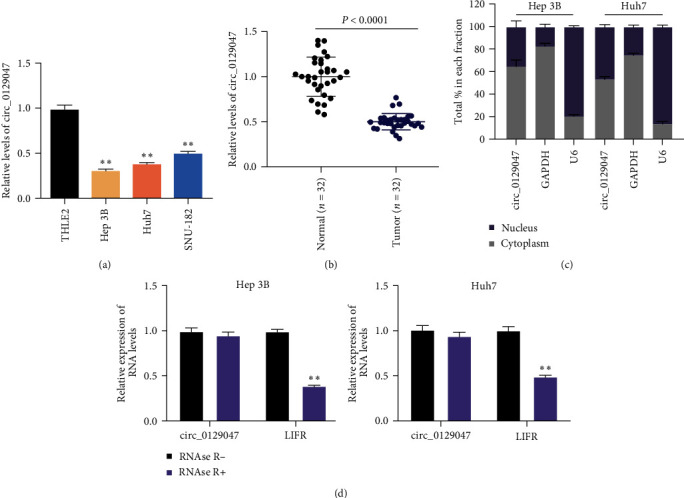
Expression of circ_0129047 decreases during HCC progression. (a) RT-qPCR analysis of circ_0129047 in HCC cells (Huh7, Hep 3B, and SNU-182) and human liver cell line THLE2. ^*∗∗*^*P* < 0.001, vs. THLE2. (b) RT-qPCR analysis of circ_0129047 in HCC and normal tissues. (c) RT-qPCR analysis of circ_0129047 in the nuclear-cytoplasmic fractionation of Huh7 and Hep 3B cells. (d) RT-qPCR analysis of circ_0129047 and LIFR (the linear gene of circ_0129047) in Huh7 and Hep 3B cells with and without RNAse R treatment.  ^*∗∗*^*P* < 0.001, vs. RNAse R.

To investigate the importance of circ_0129047 in hepatocellular carcinogenesis, we first evaluated the expression of circ_0129047 in HCC (Huh7, Hep 3B, and SNU-182) and THLE2 cells. We observed that circ_0129047 was expressed at lower levels in HCC cells than in THLE2 cells (Figure 1(a)). Among the three HCC cell lines, the expression of circ_0129047 was much lower in Huh7 and Hep 3B cells than in SNU-182 cells; therefore, we chose both HCC cell lines for subsequent assays. Constitutive downregulation of circ_0129047 was also detected in HCC tissues in contrast to normal tissues (Figure 1(b)). Subsequently, we further defined the characteristics of circ_0129047 by determining its localization and found that circ_0129047 was predominantly enriched in the cytoplasms of Huh7 and Hep 3B cells (Figure 1(c)). Furthermore, RNase A treatment had no effect on circ_0129047 expression in Huh7 and Hep 3B cells (Figure 1(d)). These findings suggested that circ_012904 plays a role in HCC progression.

### 3.2. Circ_0129047 Overexpression Delays In Vitro and In Vivo HCC Growth

**Figure 2:**
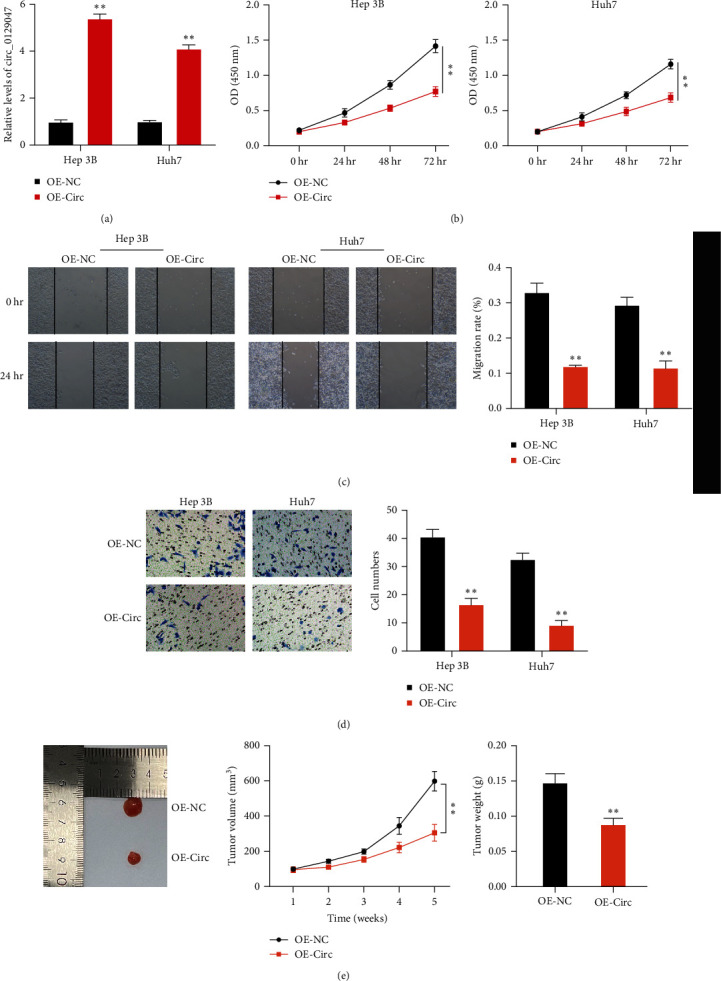
circ_0129047 overexpression delays in vitro and in vivo HCC growth. Overexpression of circ_0129047 (OE-circ) and empty vectors (OE-NC) was induced in Hep 3B and Huh7 cells. (a) RT-qPCR was verifying the exogenous expression of circ_0129047 in Hep 3B and Huh7 cells. (b) CCK8 assays examining the proliferation of Hep 3B and Huh7 cells transfected with OE-circ and OE-NC. (c) Scratch migration assay for examining HCC cell migration. (d) HCC cell invasion ability was detected using a transwell invasion assay. (e) Hep 3B cells overexpressing circ_0129047 or NC were introduced into nude mice through subcutaneous flank injection. Tumor volumes were recorded every 7 days, and xenograft tumors were excised and weighed at 5 weeks. The xenograft tumors were photographed, measured, and weighed.  ^*∗∗*^*P* < 0.001, vs. OE-NC.

To investigate whether circ_0129047 affects HCC progression, we introduced circ_0129047-overexpressing vectors or empty vectors into Huh7 and Hep 3B cells and detected the exogenous expression of circ_0129047. As expected, the upregulation of circ_0129047 was detectable in Huh7 and Hep 3B cells (Figure 2(a)), verifying the transfection efficiency. Subsequently, the cellular functional assessment of proliferation, migration, and invasion was conducted. We observed a significant reduction in the proliferation of HCC cells when circ_0129047 was overexpressed (Figure 2(b)). Scratch assays showed that the exogenous expression of circ_0129047 caused a significant reduction in HCC cell migration (Figure 2(c)). HCC cells overexpressing circ_0129047 showed a similar reduction of HCC cell invasion (Figure 2(d)). To further validate the role of circ_0129047, we developed xenograft nude mice by subcutaneous injection of Hep 3B cells overexpressing circ_0129047. We found that circ_0129047 overexpression reduced tumorigenesis in Hep 3B xenografts, as evidenced by the reduced tumor weight and size (Figure 2(e)). These results support the antitumorigenic role of circ_0129047 in HCC progression both in vitro and in vivo.

### 3.3. Circ_0129047 Targets miR-492

**Figure 3:**
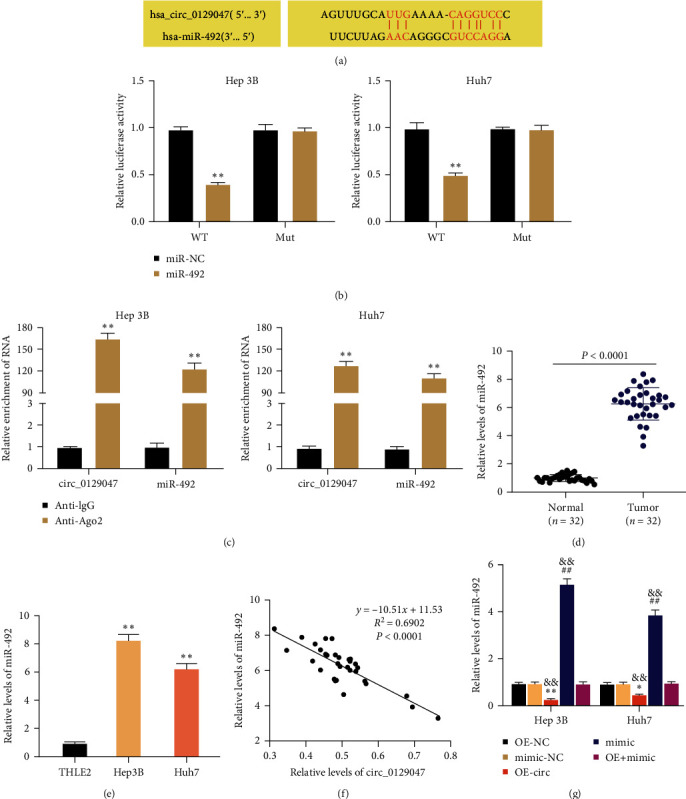
Circ_0129047 targets miR-492. (a) The binding pairs of miR-492 and circ_0129047 were predicted by CircInteractome. (b) Hep 3B and Huh7 cells were cotransfected with a miR-492 mimic or mimic NC and circ_0129047-WT or circ_0129047-MUT luciferase vectors. Luciferase activities were determined using the dual-luciferase system.  ^*∗∗*^*P* < 0.001, vs. miR-NC. (c) RT-qPCR analysis of immunoprecipitated circ_0129047 and miR-492 using an anti-Ago2 antibody.  ^*∗∗*^*P* < 0.001, vs. Anti-lgG. (d) RT-qPCR analysis of miR-492 expression in HCC tissues. (e) RT-qPCR analysis of miR-492 expression in HCC (Hep 3B and Huh7) and THLE2 cells.  ^*∗∗*^*P* < 0.001, vs. THLE2. (f) Pearson analysis of the correlation between miR-492 and circ_0129047. (g) Hep 3B and Huh7 cells transfected with OE-NC, OE-circ (OE-circ_0129047), miR-492 mimic, mimic NC, and OE-circ + mimic. The expression of miR-492 was determined by RT-qPCR at 48 hr.  ^*∗∗*^*P* < 0.001, vs. OE-NC; ^##^*P* < 0.001, vs.mimic-NC; ^&&^*P* < 0.001, vs.OE + mimic.

Cytoplasmic circRNAs serve as miRNA sponges that impair miRNA functions. Therefore, we uncovered the mechanism employed by circ_0129047 to regulate the progression of HCC. CircInteractome analysis showed that circ_0129047 is a potential target site for miR-492 (Figure 3(a)). To validate this, we assessed the luciferase activity driven by circ_0129047-WT or circ_0129047-MUT in Hep 3B and Huh7 cells upon cotransfection with the miR-492 mimic or mimic NC. We observed reduced luciferase activity in circ_0129047-WT cells when cotransfected with miR-492 mimics compared to miR-NC (Figure 3(b)). Conversely, no notable changes were observed in the luciferase activity in circ_0129047-MUT cells when cotransfected with miR-492 mimics relative to the control group (Figure 3(b)). Furthermore, circ_0129047 and miR-492 were simultaneously captured by anti-Ago2 in Hep 3B and Huh7 cells (Figure 3(c)). These data suggested that circ_0129047 targets miR-492. Furthermore, quantification of miR-492 expression using RT-qPCR revealed enhanced miR-492 expression in HCC tissues (Figure 3(d)) and cells (Figure 3(e)). Notably, a significant negative association was observed between miR-492 and circ_0129047 expression in HCC tissues (Figure 3(f)). Their relationship was further evidenced by RT-qPCR, which demonstrated that the overexpression of circ_0129047 offset the increase in miR-492 expression following miR-492 mimic transfection in HCC cells (Figure 3(g)). Thus, circ_0129047 acted as a sponge for miR-492 and reduced its expression in HCC cells.

### 3.4. miR-492 Downregulation Is Critical for the Inhibitory Effect of circ_0129047 on HCC Cell Survival

**Figure 4:**
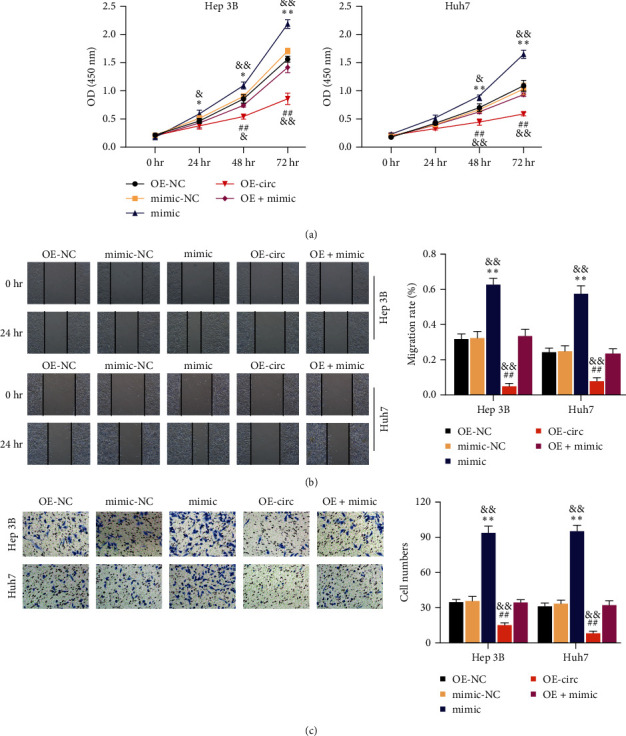
miR-492 downregulation is critical for the inhibitory effect of circ_0129047 on HCC cell survival. Hep 3B and Huh7 cells were transfected with OE-NC, OE-circ (OE-circ_0129047), miR-492 mimic, NC mimic, or OE-circ+mimic. (a) CCK8 assays were conducted to determine HCC cell proliferation. (b) Scratch migration assays were conducted to evaluate the migration rate of HCC cells after 24 hr of transfection. (c) Transwell migration assays were conducted to examine HCC cell invasion after 48 hr of transfection.  ^*∗*^*P* < 0.05;  ^*∗∗*^*P* < 0.001, vs. mimic; ^##^*P* < 0.001, vs.mimic-NC; ^&^*P* < 0.05; ^&&^*P* < 0.001, vs.OE + mimic.

After establishing the ability of circ_0129047 to regulate miR-492 expression, we investigated its function in HCC progression. We assessed the effect of cotransfection with OE-circ and miR-492 mimics on the malignant features of HCC cells. We observed enhanced HCC cell proliferation following transfection with the miR-492 mimic, which was abrogated by cotransfection with the circ_0129047 overexpression vector (Figure 4(a)). Furthermore, we detected a robust increase in the migratory rate of the miR-492-overexpressing HCC cell, whereas this higher HCC cell migration was abrogated by co-overexpression with circ_0129047 (Figure 4(b)). Additionally, miR-492 overexpression stimulated the invasive capacity of HCC cells, and when OE-circ was cotransfected, the increased invasion of HCC cells was neutralized (Figure 4(c)). In summary, circ_0129047 impairs the malignant behavior of HCC cells by sponging miR-492.

### 3.5. MiR-492 Targets LYVE1

**Figure 5:**
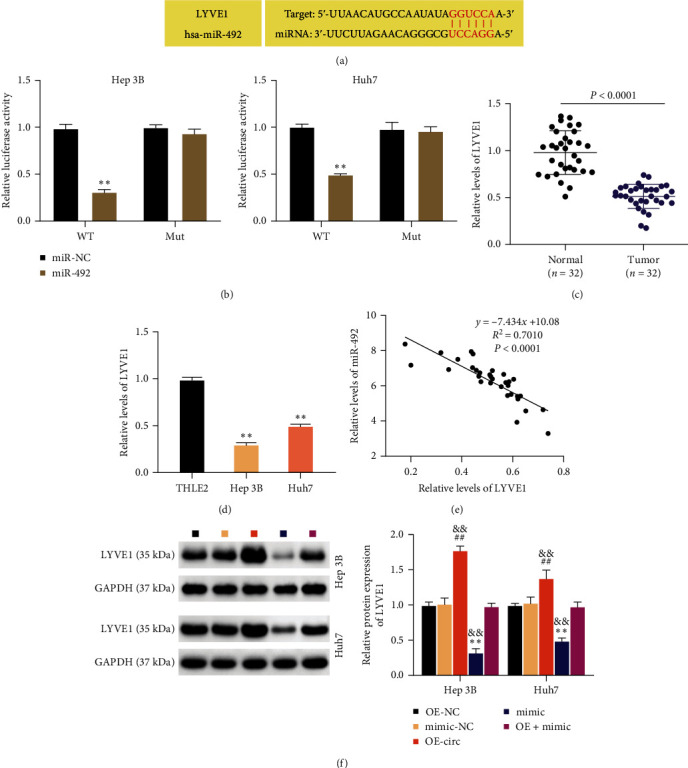
MiR-492 targets LYVE1. (a) TargetScan was used to identify the target of miR-492. (b) Luciferase activity driven by 3′UTR LYVE1 MUT and WT was assessed in Hep 3B and Huh7 cells cotransfected with miR-492 mimic or mimic NC.  ^*∗∗*^*P* < 0.001, vs. miR-NC. (c) RT-qPCR analysis of LYVE1 expression in HCC and normal tissues. (d) RT-qPCR analysis of LYVE1 in THLE2 and HCC cells.  ^*∗∗*^*P* < 0.001, vs. THLE2. (e) Pearson analysis of the association between LYVE1 and miR-492 in HCC tissues. (f) Western blotting was conducted to examine the LYVE1 expression in Hep 3B and Huh7 cells transfected with OE-NC, mimic NC, OE-circ mimic, or OE-circ + mimic.  ^*∗∗*^*P* < 0.001, vs. OE-NC; ^##^*P* < 0.001, vs.mimic-NC; ^&&^*P* < 0.001, vs.OE- circ + mimic.

Based on these data, we explored the mRNA implicated in circ_0129047-driven ceRNA activity, wherein miRNAs exert their effects by modifying the mRNA expression. TargetScan analysis predicted a binding site between miR-492 and 3′UTR LYVE1 (Figure 5(a)). To validate the interaction between them, the 3′UTR LYVE1 MUT and WT luciferase reporter vectors were created and introduced into Hep 3B and Huh7 cells accompanied by miR-492 mimic or mimic NC. Quantification of luciferase activity showed that miR-492 mimic suppressed the 3′UTR LYVE1 WT-mediated luciferase activity but had no impact on the 3′UTR LYVE1 MUT-mediated luciferase activity (Figure 5(b)). The expression of LYVE1 in HCC tissues and cells was reduced compared to that in the respective controls (Figures 5(c) and 5(d)). Furthermore, a clear negative association was observed between the expression of miR-492 and LYVE1 in HCC tissues (Figure 5(e)). Notably, the miR-492 overexpression caused an abrogation of LYVE1 expression in Hep 3B and Huh7 cells, and the circ_0129047 overexpression induced an increase in LYVE1 expression (Figure 5(f)). Moreover, the elevated LYVE1 expression caused by circ_0129047 overexpression was reversed by the miR-492 mimic. Overall, miR-492 negatively affects LYVE1 expression through recognition of 3′UTR sequences.

### 3.6. LYVE1 Overexpression Abrogates the miR-492 Oncogenic Behaviors

**Figure 6:**
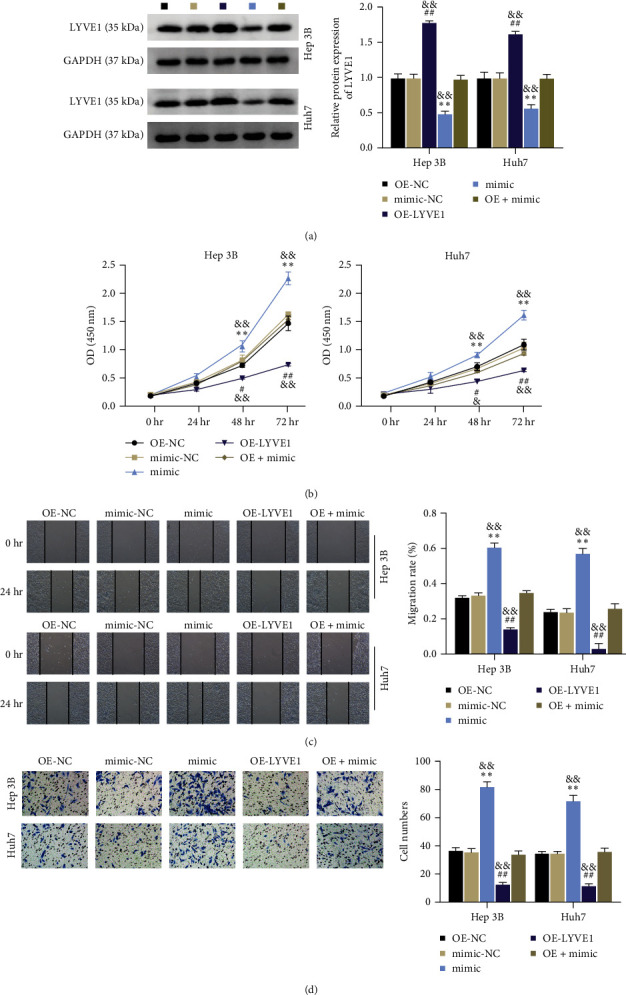
LYVE1 downregulation contributes to the oncogenic behaviors of miR-492. Hep 3B and Huh7 cells were transfected with OE-NC, mimic NC, OE-LYVE1 mimic, or OE-LYVE1+mimic. (a) Western blot conducted to examine LYVE1 expression in Hep 3B and Huh7 cells. (b) The CCK8 assay was conducted to determine HCC cell proliferation. (c) Scratch migration assays were conducted to evaluate the migration rate of HCC cells after 24 hr of transfection. (d) Transwell migration assays were conducted to examine HCC cell invasion after 48 hr of transfection.  ^*∗∗*^*P* < 0.001, vs.OE-NC; ^#^*P* < 0.05; ^##^*P* < 0.001, vs. mimic-NC; ^&^*P* < 0.05; ^&&^*P* < 0.001, vs. OE + mimic.

Subsequently, we examined the effects of miR-492 on LYVE1 expression. We observed that miR-492 overexpression caused an abrogation of LYVE1 overexpression in Hep 3B and Huh7 cells, implying that miR-492 regulates LYVE1 expression (Figure 6(a)). We investigated the role of LYVE1 in the oncogenic behavior of miR-492. Hence, we examined HCC cell phenotypes after cotransfection with the miR-492 mimic and LYVE1 overexpressing vectors. LYVE1 overexpression suppressed HCC cell proliferation, whereas this antiproliferative effect was offset by the simultaneous overexpression of LYVE1 and miR-492 (Figure 6(b)). Consistently, LYVE1 overexpression delayed HCC cell migration and invasion, and this effect was offset by the miR-492 mimic (Figures 6(c) and 6(d)). These findings indicated that LYVE1 is a key downstream effector of miR-492, which promotes malignant HCC phenotypes.

## 4. Discussion

HCC is the most prevalent and aggressive primary liver cancer [20]. Although the increase in its detection and reported incidence warrants the effectiveness of the HCC intervention before the occurrence of metastasis, the 5-year survival rate of HCC remains under 30% [20]. Therefore, there is an urgent need to fully uncover the mechanism behind HCC progression. In this study, we demonstrated the role of circ_0129047 in HCC progression. Our findings demonstrated that circ_0129047 enhances LYVE1 expression and suppresses HCC development by sponging miR-492. Our data indicated that circ_0129047 may be a viable target for HCC treatment.

Previous studies have shown that circRNAs play important roles in various biological processes in multiple malignancies, including HCC [21]. For example, circRNA-SORE triggers the malignant potential of AKT, Raf1, ERK, c-Myc, and TGF-*β*1 signaling pathways, disrupting the HCC sorafenib sensitiveness [22]. Similarly, circRNA_104075 strengthens the HCC tumorigenic potential by increasing the expression of the yes-associated protein (Yes1-associated transcriptional regulator), which is a key effector of the oncogenic Hippo signaling pathway [23]. Therefore, deciphering how circRNA interference affects HCC development is crucial. In the present study, we found that circ_0129047 is downregulated in HCC tissues and cells (Figure 1). According to previous studies, circ_0129047 was reported to play an antitumorigenic role in lung adenocarcinoma [11, 24]. However, the role of circ_0129047 in HCC remains unclear. In our study, we used gain-of-function assays to demonstrate that circ_0129047 overexpression diminished HCC cell proliferation, migration, and invasion (Figure 2). Additionally, the volume and weight of xenograft tumor-bearing Hep3B cells were attenuated by circ_0129047 overexpression (Figure 2(e)). Consequently, our findings demonstrated, for the first time, the antitumorigenic potential of circ_0129047 in HCC progression in vitro and in vivo.

Notably, cytoplasmic circRNAs compete for miRNA binding and modulate mRNA turnover [25]. circ_0129047 suppresses the progression of lung adenocarcinoma by binding to miR-375 [24] or miR-1206 [11]. In contrast to previous studies on lung adenocarcinoma, we found that circ_0129047 recognizes miR-492 and downregulates its expression in HCC cells (Figure 3). Considering miR-492, its procarcinogenic action has been observed in different cancers, such as breast cancer [26], prostate cancer [27], and ovarian cancer [28], among others. In liver cancer, the G>C polymorphism of miR-492 in HCC patients reduces the risk of death and is defined as a prognostic biomarker for HCC patients undergoing surgical resection [29]. In addition to the diagnostic and prognostic properties of miR-492, von Frowein et al. [30, 31] found that miR-492 confers increased tumorigenic and metastatic potential in hepatoblastoma. However, detailed mechanisms underlying HCC remain unclear. Our data showed that miR-492 expression was upregulated in HCC tissues and cells (Figure 3). Furthermore, in-vitro assays showed that miR-492 overexpression enhanced the malignant phenotype of HCC cells. Hence, consistent with the results of previous studies, miR-492 acts as an oncomiR in HCC cells. However, we showed, for the first time, that the promoting effect of miR-492 overexpression in HCC cells was abrogated by circ_0129047 overexpression (Figure 4). Therefore, we conclude that circ_0129047 downregulates miR-492 expression and attenuates the oncogenic activity of miR-492 in HCC.

LYVE1 is a lymphatic endothelial marker and plays a pivotal role in immunity and homeostasis [32]. Furthermore, LYVE1 reportedly controls migratory and metastatic phenotypes in cancers, which account for approximately 90% of tumor-associated deaths [19]. LYVE1 was reported to be regulated in HCC liver tissues, and a decrease in LYVE-1 expression was associated with the evolution of HCC nodules from good to poor [33]. Colombat et al. [34] used RT-qPCR to detect the expression of 31 selected genes in normal livers, cirrhotic nodules, and HCC and found that LYVE1 expression was downregulated in HCC compared with that in the normal liver. Our data showed that LYVE1 was poorly expressed in HCC tissues and cells (Figure 5), which was consistent with the results of previous studies. However, previous studies have not confirmed the effects or mechanism of action of LYVE1 on HCC progression. This study reveals the antioncogenic effects of LYVE1 in HCC. Specifically, we identified LYVE1 as a target gene of miR-492, and its downregulation caused by miR-492 overexpression in HCC cells was partially offset by circ_0129047 overexpression (Figure 5), demonstrating the involvement of the circ_0129047/ miR-492/ LYVE1 axis in HCC. Owing to their targeted relationship, the positive effect of LYVE1 overexpression on HCC cell malignancy was reversed by miR-492 overexpression.

However, our study had certain limitations that should be addressed in future studies. First, the small number of samples may have caused a selective bias. Furthermore, circ_0129047 may be responsible for trafficking different miRNAs that influence HCC progression through base-pair recognition, warranting further investigation. However, further in-vivo assays are required to corroborate our in vitro findings.

## 5. Conclusion

In conclusion, circ_0129047 expression was reduced in HCC tissues and cells, and circ_0129047 overexpression delayed HCC growth in vitro and in vivo. Moreover, circ_0129047 reduced miR-492 expression and prevented miR-492 from inhibiting LYVE1 expression, thereby promoting the development of HCC.

## Figures and Tables

**Table 1:** Real-time PCR primer sequences.

Gene name	Sequence
hsa_circ_0129047	Forward 5′-ATTCCAGCTCTTTCACATGG-3′ Reverse 5′-ATCCAGGATGGTCGTTTCAA-3′
LYVE1	Forward 5′-GCCGACAGTTTGCAGCCTATTG-3′ Reverse 5′-CCGAGTAGGTACTGTCACTGAC-3′
miR-492	Forward 5′-GCCGAGAGGACCTGCGGGA-3′ Reverse 5′-CTCAACTGGTGTCGTGGA-3′
GAPDH	Forward 5′-ATGCCTCCTGCACCACCAACTGCTT-3′ Reverse 5′-TGGCAGTGATGGCATGGACTGTGGT-3′
U6	Forward 5′-CTCGCTTCGGCAGCACA-3′ Reverse 5′-AACGCTTCACGAATTTGCGT-3′

## Data Availability

All data generated or analyzed during this study are included in this article.
